# Action 3:30R: protocol for a cluster randomised feasibility study of a revised teaching assistant-led extracurricular physical activity intervention for 8- to 10-year-olds

**DOI:** 10.1186/s40814-017-0213-0

**Published:** 2017-12-06

**Authors:** Byron Tibbitts, Alice Porter, Simon J. Sebire, Chris Metcalfe, Emma Bird, Jane Powell, Russell Jago

**Affiliations:** 1 Centre for Exercise, Nutrition & Health Sciences, School for Policy Studies, University of Bristol, 8 Priory Road, Bristol, BS8 1TZ UK; 2 Bristol Randomised Trials Collaboration, University of Bristol, Bristol, UK; 3 Health and Social Sciences, University of the West of England, Bristol, UK; 4 The National Institute for Health Research Collaboration for Leadership in Applied Health Research and Care West (NIHR CLAHRC West), University Hospitals Bristol NHS Foundation Trust, Bristol, UK

**Keywords:** Children, Physical activity, Intervention, Teaching assistant, Feasibility trial

## Abstract

**Background:**

Approximately half of 7-year-old children do not meet physical activity (PA) recommendations. Interventions targeting primary school children’s afterschool discretionary time could increase PA. Teaching assistants (TAs) are a school resource and could be trained to deliver after-school PA programmes. Building on earlier work, this paper describes the protocol for a cluster randomised feasibility study of a teaching assistant-led after-school intervention aimed at increasing PA levels of year 4 and 5 children (8–10 years old).

**Methods:**

Phase 1—pre-baseline: 12 schools will be recruited. In all schools, self-reported PA will be measured in all consenting year 3 and 4 children. In four schools, pupils will additionally wear a waist-worn Actigraph accelerometer for 7 days.

Phase 2—baseline: schools will be randomised to one of two enhanced recruitment strategies being tested for children: (1) a club briefing and (2) the briefing plus a taster Action 3:30 session. Up to 30 children per school will be able to attend Action 3:30 sessions and will provide baseline data on height, weight, psychosocial variables and accelerometer-measured PA.

Phase 3—intervention and follow-up: Schools randomised into intervention or control arm. Intervention schools (*n* = 6) will receive a 15-week after-school programme when children are in years 4 and 5, run by TAs who have attended a 25-h Action 3:30 training programme. Control schools (*n* = 6) will continue with normal practice. Follow-up measures will be a repeat of baseline measures at the end of the 15-week intervention.

Phase 4—process evaluation: session attendance, perceived enjoyment and perceived exertion will be assessed during the intervention, as well as the economic impact on schools. Post-study qualitative assessments with TAs, school contacts and pupils will identify how the programme could be refined. Accelerometer-determined minutes of moderate-to-vigorous physical activity (MVPA) per day will be calculated as this is likely to be the primary outcome in a future definitive trial.

**Discussion:**

The Action 3:30 cluster randomised feasibility trial will assess the public health potential of this intervention approach and provide the information necessary to progress to a definitive cluster randomised controlled trial.

**Trial registration:**

ISRCTN34001941. Registered 01/12/2016.

## Background

Physical activity has been shown to reduce the adult risk of heart disease, stroke, type 2 diabetes mellitus, some cancers and obesity and is associated with higher levels of mental well-being [1]. Physical activity in childhood is also associated with lower levels of a number of risk factors, including insulin, glucose, blood pressure and body composition [2], and with improved emotional well-being and self-esteem [3]. Despite the benefits of regular physical activity, objectively measured UK physical activity data show that 51% of 7-year-olds do not met the recommendation of an hour of moderate-to-vigorous intensity physical activity (MVPA) per day [4]. Whilst physical activity during childhood has been shown to moderately track into adulthood [5], physical activity levels typically decline with age [6], with the end of primary school being a key period of decline [7]. Stimulating children’s interest in, and increasing their feelings of competence in relation to, being physically active at this point could ameliorate this decline.

Schools provide opportunities to implement public health interventions to large numbers of children [8]; however, physical activity during the primary school curriculum is limited to 2 h of physical education (PE) per week. As a result, the school curriculum provides restricted opportunities for children to meet PA guidelines or develop their physical skills adequately during this key period of motor coordination and skill acquisition. Therefore, extending and maximising the quality of the current provision is likely to be a cost-effective means of increasing physical activity [9].

Time after-school is a key period that could be utilised to promote physical activity [10]. Children who are inactive after school are less likely to meet physical activity guidelines [11]; therefore, maximising the activity opportunities after-school could be an effective means of increasing physical activity in primary school children [8, 12]. Many children already participate in extra-curricular programmes for additional academic support, music, art-based activities and competitive sports [13]. Provision may be enhanced by the UK government announcement in the 2016 Budget that the primary school PE premium (which often funds after-school programmes) will be doubled to £320 million from September 2017 [14]. However, current extra-curricular provision is dominated by fee-paying provision from external practitioners, such as football coaches [15]. In the current economic climate, more cost-effective means of delivering these programmes, such as the use of existing school staff, are required.

Teaching assistants (TAs) support schools and students in many ways. Many TAs would welcome an opportunity to deliver after-school activities but lack the skills and confidence to do so. Head-teachers are keen to allow TAs to deliver after-school activities because (a) it is consistent with the Extended Schools and NICE guidance [16]; (b) commercial activity session providers are expensive [15]; and (c) it shows that the school is developing the skills of its workforce. Training TAs is an intervention approach which, if shown to be effective, could be rolled out nationwide and could be sustainable within existing school systems.

We recently completed an evaluation of the original Action 3:30 afterschool intervention [12]. The original feasibility study was conducted in 20 schools and participants were Year 5 and 6 pupils (9 to 11 year olds). Results showed that the Action 3:30 intervention could be implemented as planned, was liked by schools, children and TAs, and holds promise as a scalable physical activity approach [17, 18]. The adjusted difference in weekday MVPA at the end of the intervention was 4.3 min higher (95% CI = − 2.6 to 11.3) in the intervention arm. Exploratory, analyses indicated that the original intervention may hold more promise to increase boys’ activity (8.6 min, 95% CI = 2.8 to 14.5) than girls’ (0.15 min, 95% CI = − 9.7 to 10.0). The effect on mean levels of MVPA among boys is among the best that have been shown for physical activity interventions in children [19]. However, more work is needed to improve the content for girls, recruit less active participants, improve attendance and increase the TAs’ ability to manage disruptive behaviour [17, 18].

The aim of this study is to test the feasibility of the revised version of Action 3:30 which has been reworked to more successfully appeal to and engage girls and recruit less active children. Specifically, we have the following objectives:

- Objective 1: Optimise the intervention to increase activity in boys and girls.
- Objective 2: Identify effective means of recruiting low-active children.
- Objective 3: Assess intervention fidelity.
- Objective 4: Estimate the effect of allocation to the Action 3:30 intervention on weekday MVPA of participants and related physical activity behaviours.
- Objective 5: Collect the information needed to assess the feasibility of conducting a definitive trial and assess the implementation potential of the intervention.
- Objective 6: Assess whether progression criteria for conducting a definitive trial are met (see below).

## Methods

### Study design

Action 3:30 is a cluster randomised feasibility trial with school as the unit of allocation. Twelve schools will be recruited, with eight primary schools from South Gloucestershire local authority and four from North Somerset local authority. Schools will be recruited to provide variation in school size and the percentage of pupils eligible for free school meals (an indicator of deprivation). Participants will be pupils who are in years 3/4 (aged 7–9) at the baseline assessment in each of the schools. Data will be collected at two time-points: baseline (T0) which will take place between May and July 2017 and then during the last 6 intervention sessions (T1) which will be in February of the next academic year, meaning pupils will be in years 4/5 (aged 8–10) during the intervention and T1 data collection. The study design also includes an opt-out phase (phase 1) which will occur prior to T0 and will be used to compare the activity levels of children who consent to join the study compared to the overall sample of eligible children. Each of these phases is outlined below.

#### Phase 1: opt-out phase (pre-baseline)

All children in years 3 and 4 in the 12 study schools will be asked to complete the Physical Activity Questionnaire for Older Children (PAQ-C) scale [20] prior to the main study commencing. In four South Gloucestershire schools, children will also be asked to wear an accelerometer for 7 days. Opt-out parental consent will be used. This will allow for analysis of differences in physical activity levels between those who are recruited to phase 2.

#### Phase 2: baseline (T0)

##### Recruitment

We aim to recruit 30 year 3/4 pupils from each school. As girls are less active than boys [21], we will aim for a minimum of 40% of the sample to be girls. All year 3 and 4 children able to regularly take part in physical education lessons will be eligible. In schools where more than 30 children consent, 30 will be randomly selected for the study.

Two methods will be trialled for phase 2 recruitment: briefing (recruitment method A) or briefing plus taster session (recruitment method B). A goal of this project is to examine which of the two approaches is most effective for recruitment, to inform a recruitment strategy in a definitive future trial. Recruitment method A will involve a short briefing in each class to explain the study and provision of student information sheets and parental study information (information sheet and parental consent form to return). Recruitment method B will involve the briefing plus a taster session. Taster sessions will be led by coaches from Bristol City Council (a collaborating partner) and will involve a 20–30-min activity session like that of the Action 3:30 after-school club. Four schools in South Gloucestershire and two in North Somerset will be randomly selected to receive the taster sessions. Written parental consent will be obtained for all participants [22]. A brief questionnaire will be sent home to parents who do not give consent for the main study to assess the reasons for not wanting their child to take part.

##### Measures

All measures will be taken at baseline (T0) prior to randomisation and again during the last six intervention sessions (T1) and will be conducted by trained members of the research team. At both time points, children will wear an ActiGraph accelerometer for seven consecutive days to assess physical activity levels. The raw accelerometer data will be integrated into 10-s intervals at point of download. Periods of ≥ 60 min of zero counts will be recorded as “non-wear time” and removed. Children will be included in analysis if they provide ≥ 3 valid days (500 min of data between 6 a.m. and 11 p.m.). Minutes in MVPA will be established for weekdays and weekends, using cut-points developed for children [23]. Accelerometer counts per minute (cpm), an indication of the volume of total activity, will also be derived. Sedentary time will also be estimated based on a cut-point of less than 100 cpm. MVPA and total activity will also be assessed during the period after-school.

At each time point, children will complete a questionnaire assessing activity-based perceptions of autonomy, relatedness, competence and enjoyment using established scales. Children will also complete the KIDSCREEN-10 [24] and Child Utility 9D [25] (CHU9D) questionnaires to assess health-related quality of life. These questionnaires will be completed on tablet devices to aid data completion.

Children’s height and weight will be measured to the nearest 0.1 cm with their shoes, coats and jumpers removed, using a portable SECA stadiometer and digital SECA scale, respectively. Body mass index (kg/m^2^) will be calculated and converted to an age- and sex-specific body mass index standard deviation score [26].

Data on socioeconomic status and ethnicity of child will be collected via parental questionnaire sent home at T0. Mode of travel to and from school will also be assessed via the parental questionnaire at T0 and T1.

**Fig. 1:**
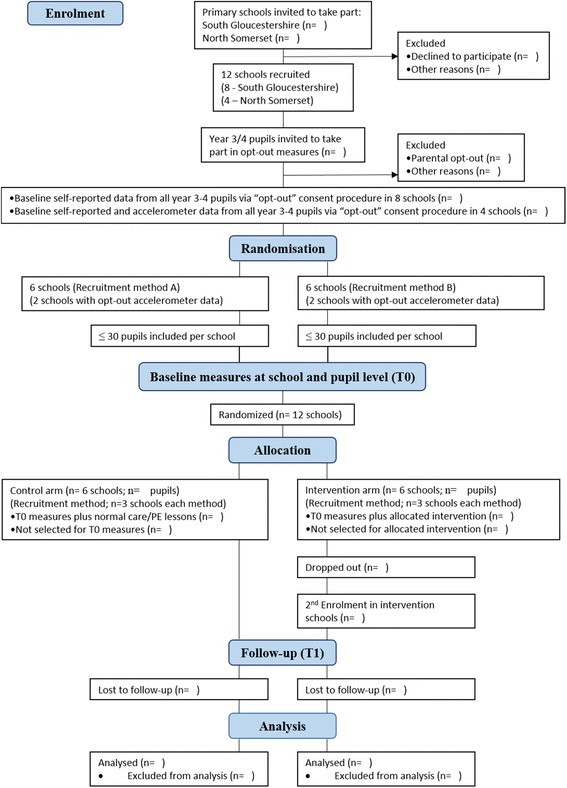
CONSORT diagram

The schedule for recruitment and proposed study measures are outlined in the SPIRIT diagram displayed in Table 1 and in the Consolidated Standards of Reporting Trials (CONSORT) diagram (Fig. 1).

**Table 1:** Action 3:30 SPIRIT diagram displaying study recruitment and measures schedule

	Study period				
	Pre-enrolment	Enrolment	Allocation	Post-allocation	Follow-up
Timepoint	Opt-out	T0	Randomization	Intervention	T1
Enrolment					
Opt-out consent	X				
Opt-out survey (12 schools)	X				
Opt-out accelerometer (4 schools)	X				X
Informed parental consent		X			
Baseline measures		X			
Randomization into study arm			X		
Interventions					
TA training (intervention schools)				X	
Extracurricular physical activity intervention (6 schools)				X	
Normal treatment (6 schools)				X	
Assessments					
Percentage opt-out	X				X
Self-reported physical activity	X				
Percentage opt-in (total, by sex)		X			
Participant characteristics		X			X
Self-reported psychosocial questionnaires		X			X
Economic evaluation				X	X
Mean daily moderate-to-vigorous physical activity		X			X
Process evaluation				X	X

##### Allocation strategy

Randomisation will take place after baseline (T0) data collection has been completed. School is the unit of randomisation. Six schools will be randomly allocated into the intervention arm and six into the control arm, stratified by local authority area (South Gloucestershire:North Somerset at a ratio of 2:1 in each arm) and recruitment method (recruitment method A:B at a ratio of 1:1 in each arm). There is potential that recruitment method B may influence children to increase their physical activity prior to baseline measurements; hence, equal numbers of taster session schools will be allocated to control and intervention arms so that any effects of the taster sessions would be balanced between the two arms and can be separated from intervention effects. Allocation will be performed (computer-generated allocation) by an independent member of the Bristol Randomised Trials Collaboration who will be blind to the school identity.

#### Phase 3: the Action 3:30 intervention and T1 data collection

Each intervention school will receive the Action 3:30 after-school club for a total of 15 weeks. The intervention will run between November and March, when the children are in years 4/5. To minimise the displacement of existing after school provision intervention, schools will be encouraged to run the two weekly sessions whenever it is most convenient to do so in the timetable, providing it takes place straight after school.

To deliver the intervention, two TAs from each intervention school will attend a 25-h (5-day) training programme focussing on delivering a physical activity programme after-school. Action 3:30 is based on principles derived from Self-Determination Theory (SDT) [18, 27], and as such, the programme will focus on promoting children’s perceptions of autonomy, belonging and competence in relation to physical activity. Among a range of techniques to promote autonomy, TAs will be encouraged to provide choice within the activities, such as leading warm-ups and adapting games and in regard to the speed at which activities progress. TAs will be trained to support competence by setting progressive activities targeting quick success balanced by optimal challenge. They will give specific praise for attempts as well as outcomes. Relatedness will be supported through empathetic TA-child interactions. Our previous study showed that TAs are able to deliver Action 3:30 in line with these strategies and the revised version of the TA training aims to clarify misunderstandings and common problems.

The 5-day training programme will be delivered by the Coach Development Manager at Bristol City Council. Once trained, TAs will deliver Action 3:30 twice a week for 15 weeks. Each session will last 60 min. Thirty detailed session plans have been produced and TAs will be asked to deliver sessions in the prescribed order. The session plans include a range of activities and games and emphasise participation and enjoyment. For 22 of the session activities, there is a video recording of model delivery; on 19 session plans, there are links to additional online resources; and on all 30, there are reminders for the TAs on how to embed the core principles of SDT within the session. Pupils will additionally be provided with 10 home activity cards that will be distributed after every three sessions. The cards reinforce session content and provide advice on how children can practice activities that have been taught during the sessions with family and friends.

##### T1 data collection

Follow-up (T1) measures, identical to those conducted at baseline (T0), will be conducted with the same children measured at baseline during the final 3 weeks of the intervention period in each intervention and control school. In those four schools where all children in year 3/4 wore accelerometers in the opt-out consent phase, this will also be repeated at T1 (now year 4/5). Details of how these data will be used are in the “Quantitative analysis” section of this paper.

##### School and pupil appreciation

Intervention schools will receive TA training and £200 worth of equipment to use for the Action 3:30 club. Schools will be remunerated for 2 h each week at an appropriate TA rate to cover the delivery cost. Control schools will receive a £300 donation to the school fund in recognition of their participation. All children who wear accelerometers at the ‘opt-out’ phase and all children who take part in the T0 and T1 measures will receive a small thank-you gift at each time point. The gift will be selected to promote physical activity and encourage prompt return of the accelerometers.

##### Re-enrolment

Data from the original Action 3:30 study and a recent evaluation of an extra-curricular dance programme highlighted that normally schools provide an opportunity for pupils who did not register an interest at the start of an after-school programme to join at a later date [12, 17, 28]. To enhance external validity, we will assess attendance rates in the intervention schools at the end of the first school term (December, 2017). If children have dropped out, we will re-enrol children who consented to the main study but did not get selected and carry out T0 measures with them before the club starts again in the second school term (January 2018). These children will also therefore be measured at T1.

#### Phase 4: process evaluation and economic evaluation

##### Process evaluation

The number of schools approached and proportion recruited will be recorded. After each Action 3:30 intervention session, TAs will be asked to record attendance and dose of the intervention (whether sessions were delivered as planned), using a log book. A member of the project team will observe three randomly-selected sessions in each intervention school to assess whether the core components of the sessions were delivered as planned. TAs will report their self-efficacy to deliver physical activity sessions (including behaviour management) before and after the TA training and after each of the observed sessions. The original scale was developed for physical education teachers [29] and has been adapted to the teaching assistant context.

The intervention participants will be asked to complete a brief perceived enjoyment questionnaire during each of the observation visits. School physical activity context will be assessed during one of the observation visits; school-level physical activity provisions will be measured using a validated school physical activity environment scale [30], and information about school physical activity policy context will be collected via an interview with the key contact, supported by a validated school policy scale [31]. School physical activity context is being measured so that any differences in social/physical environment and school policy strategies which might impact the delivery of an intervention (or how the participants might respond to it) can be examined.

The Coach Development Manager at Bristol City Council will be asked to take part in an interview, focusing on the delivery of the training and whether it could be improved. They will also be asked to complete a checklist to assess whether TA training was delivered as planned.

Following completion of the 15-week Action 3:30 programme, all intervention TAs (*n* = 12) and intervention school contacts (*n* = 6) will be asked to take part in a semi-structured interview, and children (*n* = 6 boys, *n* = 6 girls per intervention school) will be asked to take part in focus groups. The interviews and focus groups will examine factors that may have affected recruitment, attendance, delivery and enjoyment and will ask for opinions on how these elements could be improved. Child focus groups will include questions relating to whether children’s perceptions of autonomy, belonging and competence were supported by the intervention and their interactions with the TAs. TAs will also be asked about the effectiveness of managing disruptive behaviour, their training and whether it could be improved. School contacts (staff members who act as the primary liaison between the school and the study team) will be asked about the long-term sustainability of the Action 3:30 programme and feasibility of the re-enrolment stage.

Interviews will be conducted with school contacts and class teachers in the four schools that took part in the accelerometer opt-out process. The interviews will focus on school burden and how it could be reduced, logistical issues and how the opt-out consent process could impact participation in a future trial.

Interviews (*n* = 8) will be conducted with leaders of school consortium groups, common in the English education system and local public health commissioners to discuss the sustainability and potential future dissemination of the intervention.

##### Economic evaluation

We will ask all 12 schools to report number, cost and funding source of all their extra-curricular clubs, using a retrospective survey at T0. The project team will track the costs and resources used to prepare and deliver Action 3:30. Parental time to collect children after-school by travel mode will be estimated from pupil self-reported data. The economic assessment will be based on the RE-AIM framework [32] and will assess the potential for change in health-related quality of life (*Effectiveness*), the ability to assess costs variation in delivery among school settings (*Implementation*) and the potential to sustain the outcome of the intervention from a cost perspective (*Maintenance*).

### Analysis plan

#### Quantitative analysis

The main analysis will focus on data relating to characteristics of the sample, recruitment, retention and Action 3:30 session attendance rates, levels of data provision using appropriate descriptive statistics (M, SD, Median, N & %). Evidence of promise of the intervention (i.e., estimates of the potential effect of the intervention on weekday MVPA) will be estimated using random-effects linear regression models adjusting for intervention group, local authority, and baseline MVPA with a focus on 95% confidence intervals with exploratory subgroup analyses by child gender. We will also examine the physical activity profile of children who did not attend the Action 3:30 sessions to assess whether their baseline physical activity levels were different to children who attended.

Secondary analysis will pilot the use of a complier average causal effect (CACE) analysis [33] for the four schools that used the “accelerometer opt-out” consent procedure. This model will examine the effect of receiving Action 3:30 on MVPA (original recruits and late joiners) when compared to the control arm and will provide an indication of the overall benefits of the study for all participants who were exposed to Action 3:30 as part of the original sample and late joiners.

Potential sample sizes for a future definitive trial will be estimated using the derived intraclass correlation coefficient (ICC) for MVPA from this study and published ICCs from comparable studies, the target differences in MVPA, and combinations of key parameters (type I and type II error).

#### Qualitative analysis

All interviews and focus groups will be transcribed verbatim and anonymized. Thematic analysis techniques will be employed to produce initial codes using NVivo (QSR International Pty Ltd), which will be grouped to form themes that describe the content of codes [34]. Analysis will focus on factors that might have affected recruitment, delivery, behaviour management, attendance, enjoyment, potential improvements, contamination between study arms, and any inequalities that might affect future delivery. Using the Framework Method [35], data will be triangulated from different stakeholder sources to explore interaction between the themes identified by stakeholder groups. Child focus groups will be conducted with boys and girls separately so that the intervention can be examined for improvement in terms of appeal and suitability for both girls and boys as well as any gender-specific issues using these data.

Study analyses will be used to assess whether the study has met the following five progression criteria set by the funder to qualify for a definitive full cluster randomised controlled trial:

- 1/4 of schools that are approached agree to join the study.
- 1/4 of eligible year 4/5 pupils express an interest in the study by returning consent forms.
- At least 40% of participants expressing an interest in the study are girls.
- At least 50% of the participants in the intervention arm attend 50% of the sessions.
- At T1, at least a small benefit for weekday MVPA is observed for boys and girls, comparing intervention to control schools, and the upper bound of the 95% CI for each difference exceeds a 10-min benefit for the intervention group.

Based on discussion with our independent Trial Steering Committee and following the recent CONSORT guidance [36] for pilot and feasibility studies, we will report against these five criteria using a red, amber or green traffic light system.

## Discussion

This paper describes the protocol for a feasibility study of a new, improved iteration of the Action 3:30 intervention, which aims to increase physical activity among year 4 and 5 children in UK primary schools. Many children do not engage in a sufficient amount of physical activity, especially within the school curriculum. Primary school is a key period to establish physical activity behaviours, preferences and skills. The after-school period is an ideal time to provide physical activity provision. However, there is currently a lack of research-based well-evaluated after school interventions. The original Action 3:30 study showed that this intervention has the potential to improve the physical activity of children. However, in the original study, the intervention was more effective for boys than girls and recruitment strategies required improvement. We also identified that it would be important to identify whether it is possible to increase external validity by allowing a second enrolment of pupils. The goal of this study is to systematically test, via a feasibility study, whether addressing these issues results in a public health intervention that shows sufficient promise to warrant evaluation via a larger, cluster randomised controlled trial.

## Abbreviations

CACE: Complier Average Casual Effect
CHU9D: Child Health Utility 9D wellness scale
CI: Confidence interval
cpm: Counts per minute
ICC: Intraclass Correlation Coefficient
MVPA: Moderate to Vigorous Physical Activity
PAQ-C: Physical Activity Questionnaire for older Children
PE: Physical education
SDT: Self-Determination Theory
T0: Time point 0 (baseline)
T1: Time point 1 (follow up)
TA: Teaching assistant
